# Improvement of Biodesulfurization Rate of Alginate Immobilized *Rhodococcus erythropolis *R1

**DOI:** 10.5812/jjm.9123

**Published:** 2014-03-01

**Authors:** Peyman Derikvand, Zahra Etemadifar

**Affiliations:** 1 Department of Biology, Faculty of Sciences, University of Isfahan, Isfahan, IR Iran

**Keywords:** Immobilization, Nano particles, Alginate, Flow Cytometry, Optimization

## Abstract

**Background:**

Sulfur oxides released from the burning of oil causes severe environmental pollution. The sulfur can be removed via the 4S pathway in biodesulfurization (BDS). Immobilization approaches have been developed to prevent cell contamination of oil during the BDS process.

**Objectives:**

The encapsulation of *Rhodococcus erythropolis* R1 in calcium alginate beads was studied in order to enhance conversion of dibenzothiophene (DBT) to 2-hydroxy biphenyl (2-HBP) as the final product. Also the effect of different factors on the BDS process was investigated.

**Materials and Methods:**

Calcium alginate capsules were prepared using peristaltic pumps with different needle sizes to control the beads sizes. Scanning electron microscopy and flow cytometry methods were used to study the distribution and viability of encapsulated cells, respectively. Two non-ionic surfactants and also nano Ƴ-Al_2_O_3_were used with the ratio of 0.5% (v/v) and 1:5 (v/v) respectively to investigate their BDS efficiency. In addition, the effect of different bead sizes and different concentrations of sodium alginate in BDS activity was studied.

**Results:**

The 2% (w/v) sodium alginate beads with 1.5mm size were found to be the optimum for beads stability and efficient 2-HBP production. The viability of encapsulated cells decreased by 12% after 20 h of desulfurization, compared to free cells. Adding the non-ionic surfactants markedly enhanced the rate of BDS, because of increasing mass transfer of DBT to the gel matrix. In addition, Span 80 was more effective than Tween 80. The nanoƳ-Al_2_O_3_ particles could increase BDS rate by up to two-folds greater than that of the control beads.

**Conclusions:**

The nano Ƴ-Al_2_O_3_ can improve the immobilized biocatalyst for excellent efficiency of DBT desulfurization. Also the BDS activity can be enhanced by setting the other explained factors at optimum levels.

## 1. Background

Consumption of sulfur-rich fossil fuels leads to the release of sulfur oxides and therefore creates severe air pollution and acid rain. A major part of petroleum sulfur compounds is organic compounds and one of the main problems of oil refining is its refinement from crude oil (1). Although the physical-chemical process can remove inorganic sulfur, organic sulfur compounds such as dibenzothiophene (DBT) remain in oil after this process (2) and these resistant compounds can be removed by biodesulfurization (BDS). Due to specificity and cost-effectiveness, BDS can be an alternative or complementary process to hydrodesulfurization (HDS) technology (3). Some of the bacterial strains such as *Rhodococcus* sp. can remove sulfur from DBT and produce 2-HBP as the final product without causing oxidative loss of fuel carbon. This process is done through the 4S pathway, which is a multi-enzyme system. This system includes four genes: *dsz*A, *dsz*B and *dsz*C encoded by the plasmid, and *dsz*D encoded by the bacterial chromosome (4, 5).

Many previous studies have been conducted on BDS using whole cell (6, 7) or enzymes. Use of enzymes has disadvantages such as expensive enzyme extraction and also enzyme’s constant need to cofactors (8). Therefore, in most cases, the whole cells are used in BDS instead of enzymes. However, the use of whole cells is also limited. For example, using free cells in BDS leads to mixture of oil with cells and a cell suspension is formed where separation of cells requires centrifugation, which has high cost. In addition, there is the possibility of cell contamination of the products (9). To solve these problems, immobilization methods are used in the industry. Compared to using free cells, immobilization has some advantages such as: enhanced stability, easy separation of cells, minimization or elimination of cell contamination of the product, and easy recovery and re-use of cells, which enables their continuous use (10).

Alginate is among biopolymers that can be employed in encapsulation of cells and enzymes and because of its advantages such as biocompatibility, simplicity and low cost, it has been widely used in industrial processes (11). Flow cytometry is one of the viability measurement methods, which very precisely enables rapid analysis of thousands of cells in a few seconds (12). Also in other procedures such as the plate counting method, some bacteria are physiologically active but unable to divide and are considered as dead cells, but the problem does not exist in flow cytometry (13). Therefore, in the current study immobilization of *Rhodococcus erythropolis* R1 by calcium alginate was done, and the strategies to improve the rate of desulfurization (such as nono-coating and surfactant) by immobilized cells were investigated. In addition, viability of entrapped cells was measured by flow cytometry.

## 2. Objectives

The aim of the current study was biodesulfurization of DBT by immobilized cells and improvement of their activity. 

## 3. Materials and Methods

### 3.1. Chemicals

Sodium alginate, rhodamine 123 and nano Ƴ-Al_2_O_3_ were purchased from Sigma Chemical Co. DBT was purchased from Merck. 2-HBP, Span 80 and Tween 80 were obtained from Fluka Chemika. All other chemicals were analytical grade and commercially available.

### 3.2. Bacterial Strain and Growth Condition

*R. erythropolis* R1 (NCBI GenBank Accession No. GU570564), a capable strain in desulfurizing DBT to 2-HBP was previously isolated from oil-contaminated soil (14) and was cultured in basal salt medium (BSM) supplemented with 0.3 mM DBT as the sole sulfur source. Cell cultivation was conducted in a 1000 mL flask containing 200 mL of BSM medium at 30°C on a rotatory shaker operated at 180 rpm (n-biotech, Inc). The BSM had the following composition: Na_2_HPO_4_.7H_2_O 8 g/L, KH_2_PO_4_ 4 g/L, NH_4_Cl 2 g/L, MgCl_2_ 0.2 g/L, FeCl_3_ 0.001 g/L, CaCl_2_ 0.001 g/L and glucose 15 g/L as the carbon source.

### 3.3. Entrapment of Bacteria

Cells were harvested after 72 h by centrifugation at 7000 rpm for 10 min, washed several times with 0.9% NaCl in order to remove DBT remaining on the surface of cells, and then resuspended to adjust the cell mass to an OD_600nm_ of 40. Sodium alginate solution was prepared by the slow addition of the alginate powder and stirring of the solution to avoid the formation of precipitation. The alginate concentrations were 2%, 4% and 6%, and were mixed well with equal volumes of cell suspension, so that the final concentrations of sodium alginate were 1%, 2% and 3%, respectively. To examine the size effect of the beads on the BDS, capsules with diameters of 1.5, 2.5 and 4 mm were prepared by different niddle sizes. The target concentration of surfactants (Tween 80 and Span 80) was 0.5% (v/v) and also volume ratio of cell mass to nanoƳ-Al_2_O_3 _was 5:1 (v/v). The alginate-cell suspension was added in a drop-wise manner to 0.2 M CaCl_2_ using a peristaltic pump and magnetic stirring to avoid droplet aggregation. Gelation time was restricted to one hour and afterwards, the beads were washed twice with modified BSM (MBSM) and then kept in MBSM supplemented with 0.1 mM DBT in order to stay active. In the MBSM composition, the value of phosphate component of BSM was reduced to 0.1 to avoid dissolution of alginate beads. Other components were the same as those in BSM.

### 3.4. Determination of Immobilized Cell Biodesulfurization Activity

The reaction solution contained immobilized cells and MBSM supplemented with 1 mM DBT and the reaction was conducted in 250 mL flasks at 30˚C and 180 rpm. After specific periods of time, 2-HBP production was measured by the Gibb's assay. After the reaction, beads were separated and kept in MBSM supplemented with 0.1 mM DBT for recycle desulfurization.

### 3.5. Cells Viability Determination

Ten individual beads were added to the dissolving buffer, mixed slowly to allow them to fully dissolve, and then washed twice with phosphate buffered saline (PBS), followed by suspension in this solution. The dissolving buffer composed of 55 mM sodium citrate, 30 mM EDTA, and 0.15 M NaCl. Rh-123 was made up to 1 mg/mL in ethanol and maintained at -20˚C as stock solution. The working concentration of Rh-123 was 10 µg/mL, which was freshly prepared in PBS on the day of the experiment. 100 µL of the cell suspension was transferred to a 5 mL polypropylene tube and 400 µL of the stain was added to it at room temperature, and then it was placed in the dark for 10 min. Measurements by the flow cytometer were performed directly thereafter (14).

### 3.6. Analytical Methods

Cell density was measured by absorbance at 600 nm (OD_600nm_). To determine the average size of beads, ten individual beads were measured with vernier calipers. Desulfurization activity was monitored using the Gibb’s reagent (2,6-dichloroquinone-4-chloroimide). Gibb’s reagent reacts with aromatic hydroxyl groups such as 2-HBP at a pH of 8.0 to form a blue-colored complex that can be monitored spectrophotometrically at 610 nm wavelength. The Gibb’s assay was done as follows: microbial culture was centrifuged at 7000 rpm for 10 min and 1 mL of supernatant was diluted, placed in a clean test tube and its pH adjusted to 8.0 by adding 200 µL of NaHCO_3_ 1 M, followed by 20 µL of Gibb’s reagent (10 mM). The solution was incubated for 30 min at room temperature to produce full-color and then the absorbance of the solution was measured at 610 nm (spectronic 21D Milton Roy). The BDS activity of the cells was determined as the percentage of desulfurization according to the following Equation 1: 

Equation 1. $$X_{\mathrm{BDS}}=\frac{C_{2-\mathrm{HBP}}}{C_{D\mathrm{BT}0}}\times 100$$

Where CDBT0 is the initial concentration of DBT (mM) and C2-HBP is the 2-HBP (mM) concentration after a certain period of time. The morphology of alginate-immobilized cells and nano Ƴ-Al_2_O_3_ on the surface of cells was determined using a scanning electron microscope (SEM) (Seron technology AIS2100). Flow cytometry was conducted using a BD FACS Calibur Flow Cytometer 342976 (USA) by a fluorescence detector that detects appropriately filtered light through the green channel (FL1, 525 nm). A total of 10,000 cells were recorded for each sample. All of the experiments were performed in duplicate.

## 4. Results and Discussion

### 4.1. Scanning Electron Microscopy of Immobilized Cells

**Figure 1.**
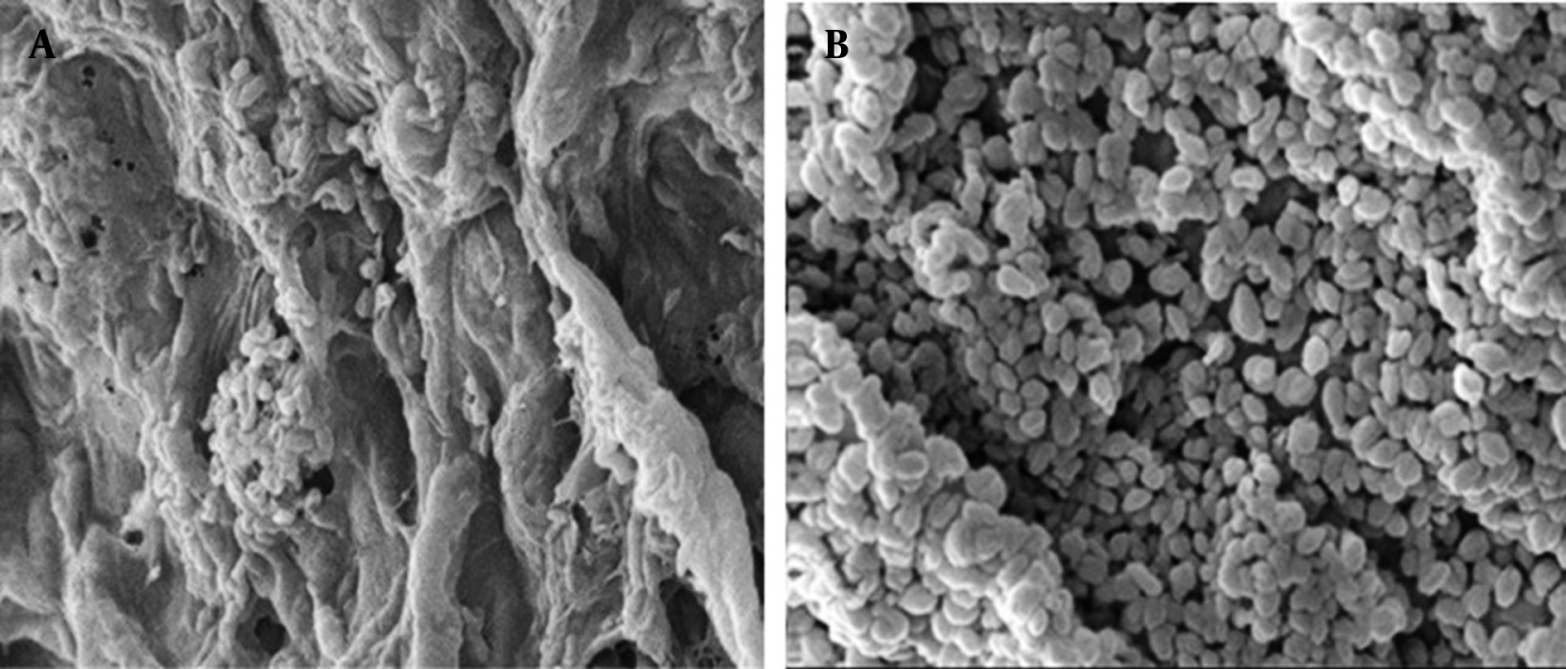
Scanning Electron Microscopy of Surface (a) and a Section (b) of an Alginate Bead

Cells distribution in alginate beads were visually evaluated by SEM observations of the surface and sections of the beads. As shown in Figure 1, only a few *R. erythropolis* R1 cells were attached to the surface of alginate beads (Figure 1a) while a large number of entrapped cells were observed in the transect of the beads (Figure 1b).

### 4.2. Effect of Bead Size on Biodesulfurization

**Figure 2.**
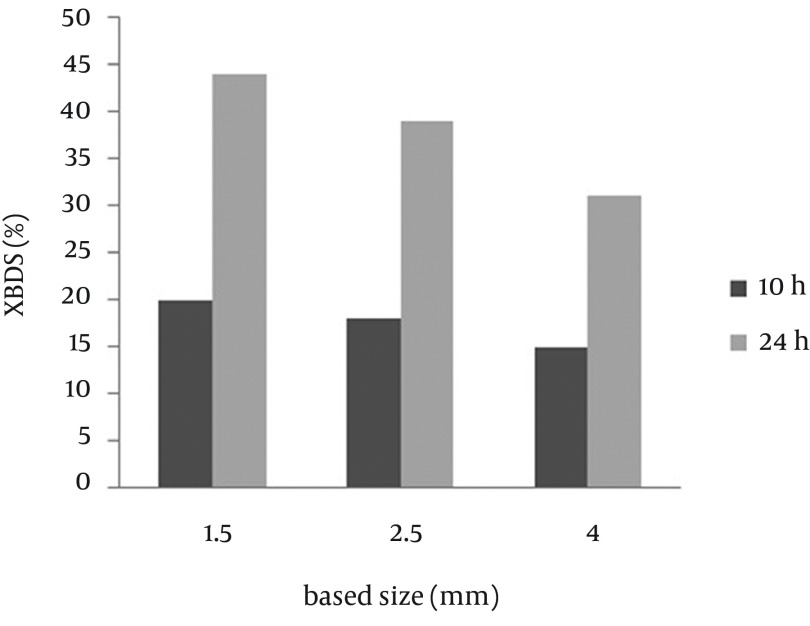
Effect of Bead Diameter on Biodesulfurization Rate of Alginate-Immobilized Cells

Small beads have high surface volume ratio, which might reduce the mass transfer limitation. Previous studies (15, 16) have indicated that reduction in the alginate bead diameter increases efficiency of the beads. To examine the size effect of the beads on the BDS, capsules with diameters of 1.5, 2.5 and 4 mm were prepared. Figure 2 shows the result of 2-HBP production after 10 and 24 h from the reaction. It was observed that bead size of alginate capsules play an important role in determination of the reaction rate. The 2-HBP production after 24 hours was 0.44, 0.39 and 0.30 mM, respectively. This result indicates that the conversion ratio of the 4 mm beads was lower than that of the 1.5 and 2.5 mm beads. According to the current study, the desulfurization rate improved with decreasing the bead size and the optimum diameter for immobilized beads was found to be 1.5 mm.

### 4.3. Effect of Alginate Concentration

**Figure 3.**
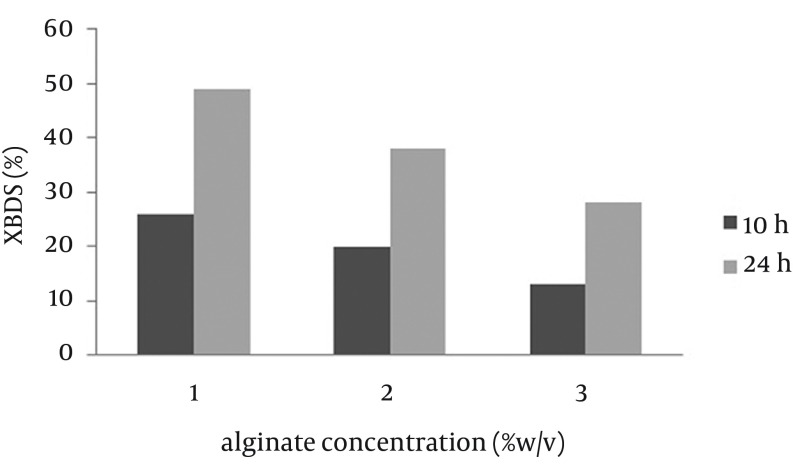
Effect of Alginate Concentration on Biodesulfurization Rate of Immobilized Cells

The stability and re-usability of beads improved with increasing alginate concentration and beads with high concentration of alginate were more spherical and could be used several times in BDS. However, due to reduction of leakage and mass transfer in alginate immobilized cells, the 2-HBP production was decreased. In contrast, beads with low alginate concentration were relatively soft and had favored leakage to mass transfer (17). Zhang et al. (18) showed that a decrease in alginate concentration leads to an increase in efficiency of beads. As shown in Figure 3, 2-HBP production after 24 hours using beads with 1% alginate concentration was 1.75 fold more than that of 3% alginate concentration. In addition, beads with 1% alginate concentration were not rigid enough and had low stability and spherical shape, thus 2% (w/v) sodium alginate concentration was determined to be the optimum concentration for efficient bioconversion.

### 4.4. Surfactant Effect on Biodesulfurization Rate

**Figure 4.**
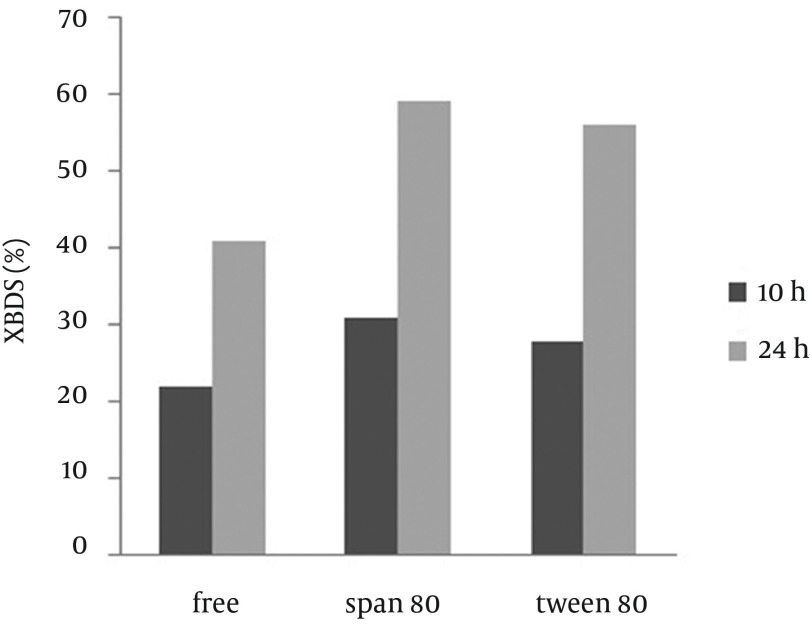
Effect of Non-Ionic Surfactants on Desulfurization Activity of Immobilized Beads

In this part of the study, the effect of two non-ionic surfactants (Tween 80 and Span 80) on DBT BDS and 2-HBP production rates were investigated. Use of surfactants, compared with the control, results greater DBT to be dissolved in the aqueous phase and allows easier penetration into beads and as a result leads to an increase of 2-HBP production. In addition, 2-HBP as a product of DBT BDS is not fully soluble in the aqueous phase and displays an inhibitory effect on biodesulfurization activity by aggregation around the cell. Feng et al. (19) showed that Tween 80 enhanced the rate of 2-HBP production. Li et al. (16) looked at the effect of different surfactants on BDS of *Pseudomonas delafieldii* and showed that Tween 80 and Span 80 had more efficiency than the others. Therefore, we studied the effect of these surfactants on alginate immobilized* R. erythropolis *R1 in comparison to other factors that affect BDS. According to the results shown in Figure 4, we concluded that the micellar solution of Tween 80 and Span 80 reduced the concentration of 2-HBP around the cells, which accounts for the increase in the desulfurization activity. In addition, Span 80 has a greater impact on BDS of DBT as compared to Tween 80, which is probably because of a greater reduction in the surface tension of the water medium.

### 4.5. Biodesulfurization of Alginate Beads Containing Cells Assembled With Nano Ƴ-Al2O3

**Figure 5.**
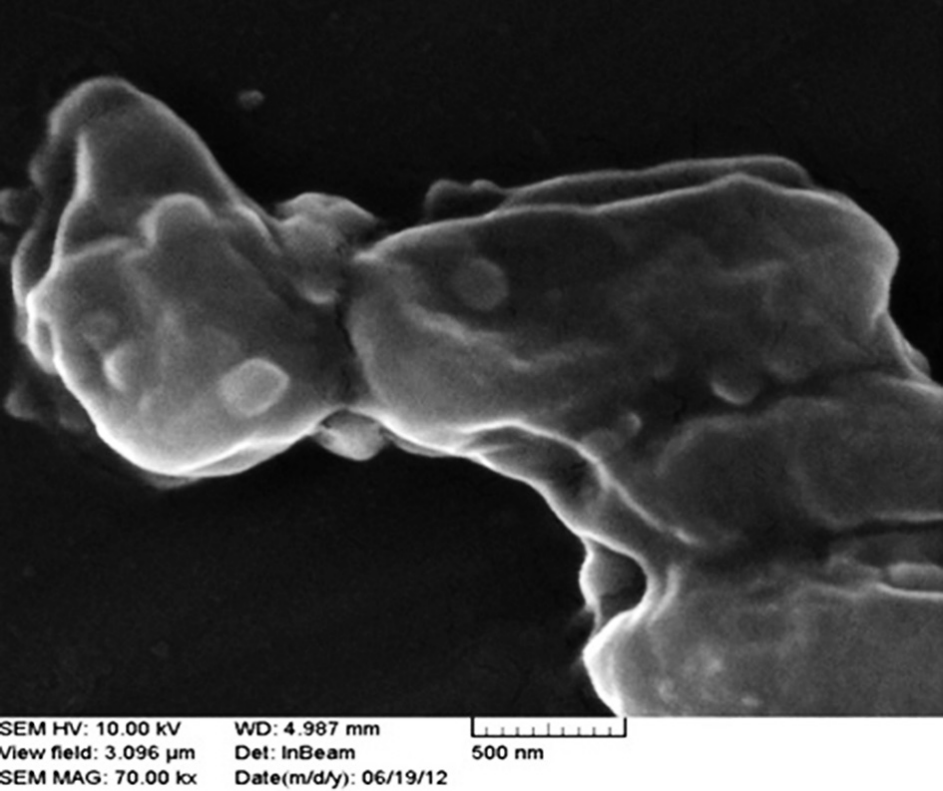
Dispersion of Ƴ-Al_2_O_3_Nano Particles on Cell

Cell surfaces are negatively charged while nano Ƴ-Al_2_O_3_is positively charged and therefore electronic force leads to nano Ƴ-Al_2_O_3_absorption on cell surfaces. Figure 5 shows the morphology of absorption on cells. Absorption of Ƴ-Al_2_O_3 _nano particles on cell surfaces leads to increasing consumption of DBT and production of 2-HBP in alginate beads and also over aggregation of absorbents on cell leads to a decrease in cell activity; a previous report (20) indicated that if particles on a cell occupy more than 2/3 of the cell surface area, this will have a negative impact on cell activity.

**Figure 6.**
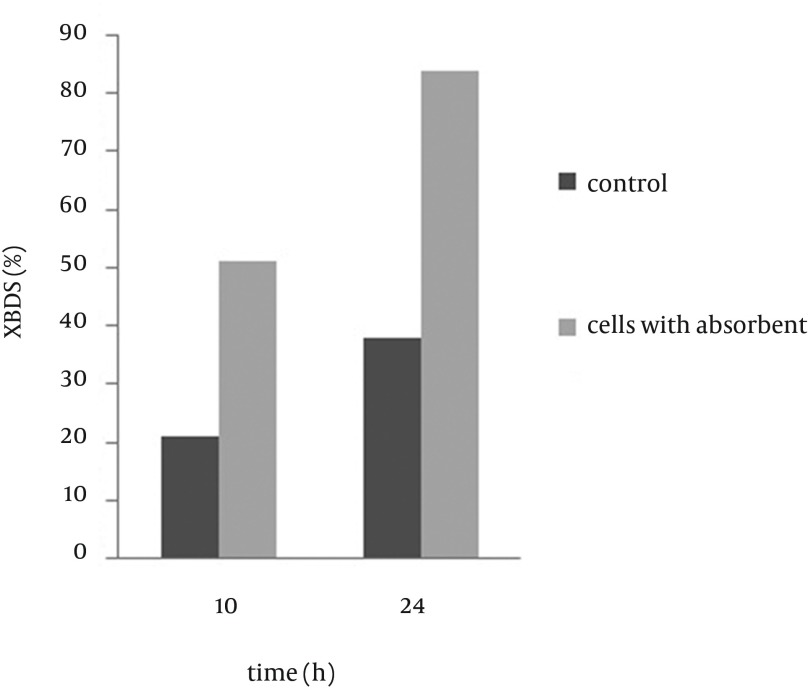
Effect of Nano Ƴ-Al_2_O_3_ on Biodesulfurization Rate of Immobilized Cells (The Control was Immobilized Cells Without Nano Particles)

**Figure 7.**
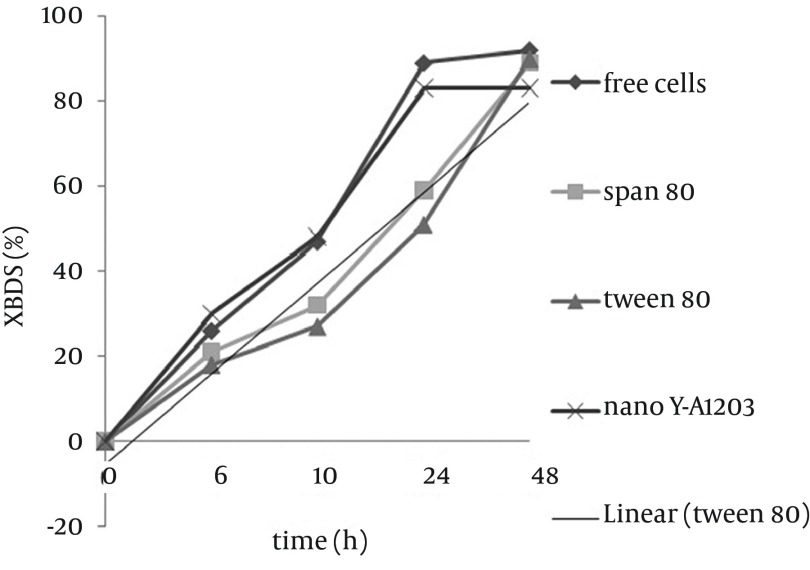
Comparison of Desulfurization Rate of Free Cells and Immobilized Cells With Span 80, Tween 80 and Nano Ƴ-Al_2_O_3_

Zhang et al. (21) showed that both DBT consumption rate and 2-HBP production rate in the coupling system of Ƴ-Al_2_O_3 _nano particles are higher than that of the free cell system and these particles are more efficient than other absorbents such as active carbon. Zhang et al. (22) used these nano particles on the surface of magnetic immobilized cells and proved that this combination leads to increase in BDS activity of immobilized cells. Figure 6 shows the comparison between alginate beads containing cells assembled with nano Ƴ-Al_2_O_3 _and control beads, and as it is illustrated, 2-HBP production by beads including cells assembled with nano Ƴ-Al_2_O_3 _after 24 hours is two folds more than that of control beads, therefore combination of nano Ƴ-Al_2_O_3 _and alginate immobilized cells in BDS can be very effective. Increase of BDS was because of specific surface area of nano Ƴ-Al_2_O_3 _and pore creation that causes more absorption of DBT on cell surface and easy transfer into the cell. Due to nano particles absorption, some of produced 2-HBP remain at the cell surface and cannot be released completely in medium. In Figure 7, illustrates the BDS rate of alginate beads including nano Ƴ-Al_2_O_3_, Span 80 and Tween 80 compared to free cells. According to the presented results, BDS rate of beads containing cells assembled with nano Ƴ-Al_2_O_3 _after 12 hours was more than that of free cells yet, final amount of 2-HBP production after 48 h was less than those of the other conditions, which was probably due to assembly of 2-HBP on the surface of nano particles. The Gibb’s reagent only reacts with aromatic hydroxyl groups and has no color reaction with Ƴ-Al_2_O_3 _or surfactants without substrate (data are not presented). It can only enhance the biodesulfurization by more exposure of substrate to bacterial cells.

### 4.6. Viability Determination

**Figure 8.**
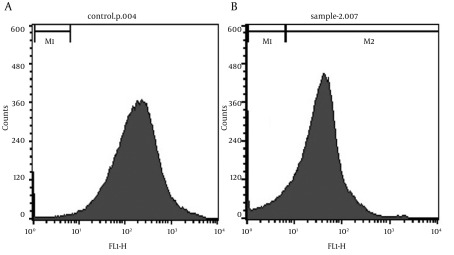
Viability of Alginate Immobilized *R. erythropolis* R1 by Flow Cytometry a. non-immobilized or free cells (positive control), b. immobilized cells after 20 hours of biodesulfurization. The M1 area shows dead cells, and viable bacteria are in the M2 area.

The inner part of the cell is negatively charged, in comparison with the outer part of the cell. Membrane potential plays a major role in various cellular processes such as: active transport, ATP synthesis, etc. Voltage-sensitive dyes have been developed to estimate membrane potential in bacteria. Rhodamine 123 is a cationic dye, which can accumulate in polarized cells. It can cross cytoplasmic membranes, but is only held inside the cells that have a negative inside-membrane potential. Viable bacteria also accumulate rhodamine 123, but non-viable bacteria cannot do so (13). Under appropriate conditions, the amount of bacteria that can accumulate rhodamine 123 quantitatively reflects the extent of their viability. As illustrated by Figure 8 a, the viability of non-immobilized cells is about 98%. By immobilization and after 20h of BDS activity, the viability decreased to about 86% (Figure 8 b), which is an acceptable value (only 12% reduction of viable cells). It can be concluded that alginate immobilization does not have a large influence on cell viability. Some of the reduction in the value of viable cells may be due to re-dissolving of beads and repeated centrifugation of cells or storage at 4˚C.

## 5. Conclusion

Improvement of biodesulfurization rate of biocatalysts is crucial for the industrialization of BDS technology in the future and reduces the air contamination and harmful effects of SO_x_, released from combustion of organic sulfur from fossil fuels, on the human health. The BDS rate can be improved by adding Span 80, layering the nano Ƴ-Al_2_O_3 _on the cell surface of biocatalyst cells, decreasing the alginate beads size, and optimizing the alginate concentration. These conditions may also be used for other biocatalysts or other biotransformation processes.
